# Association of Elevated Expression Levels of *COL4A1* in Stromal Cells with an Immunosuppressive Tumor Microenvironment in Low-Grade Glioma, Pancreatic Adenocarcinoma, Skin Cutaneous Melanoma, and Stomach Adenocarcinoma

**DOI:** 10.3390/jpm12040534

**Published:** 2022-03-28

**Authors:** Hyo-Jae Shin, Minchan Gil, Im-Soon Lee

**Affiliations:** 1 Department of Biological Sciences, Konkuk University, 120 Neungdong-ro, Gwangjin-gu, Seoul 05029, Korea; shinhyojae0606@gmail.com; 2 Department of Stem Cell and Regenerative Biotechnology, Konkuk University, 120 Neungdong-ro, Gwangjin-gu, Seoul 05029, Korea

**Keywords:** *COL4A1*, stromal cells, poor prognosis

## Abstract

Aberrant expression of collagen type IV alpha chain 1 (*COL4A1*) can influence tumor cell behavior. To examine the association of *COL4A1* expression in the tumor microenvironment (TME) with tumor progression, we performed bioinformatics analyses of The Cancer Genome Atlas RNA sequencing and RNA microarray datasets available in public databases and identified upregulated *COL4A1* expression in most examined tumor types compared to their normal counterparts. The elevated expression of *COL4A1* was correlated with low survival rates of patients with low-grade glioma, pancreatic adenocarcinoma, skin cutaneous melanoma, and stomach adenocarcinoma, thus suggesting its potential use as a biomarker for the poor prognosis of these tumors. However, *COL4A1* was mostly expressed in adjacent stromal cells, such as cancer-associated fibroblasts (CAFs) and endothelial cells. Additionally, *COL4A1* expression was highly correlated with the signatures of CAFs and endothelial cells in all four tumor types. The expression of marker genes for the infiltration of pro-tumoral immune cells, such as Treg, M2, and TAM, and those of immunosuppressive cytokines exhibited very strong positive correlations with *COL4A1* expression. Collectively, our data suggest that *COL4A1* overexpression in stromal cells may be a potential regulator of tumor-supporting TME composition associated with poor prognosis.

## 1. Introduction

Collagens are the most abundant proteins in mammals and play structural roles in supporting the mechanical properties, organization, and shape of various tissues, in addition to their regulatory roles in the physiological processes of cells, such as proliferation, migration, and differentiation, via receptors [1]. The collagen superfamily consists of 28 vertebrate types, which are distinguished by triple Gly-X-Y repeats in alpha chains forming collagen trimers [1]. *COL4A1* encodes the alpha 1 chain of type IV collagen (Col IV), which combines with alpha 1 and alpha 2 chains to form the complete IV collagen alpha 1-1-2 molecule [2]. Notably, the clinical phenotypes of patients with *COL4A1* variants are extremely variable and mutations in *COL4A1* cause a wide spectrum of conditions called “*COL4A1*-related disorders” with eye defects, cerebral small vessel disease with or without ocular anomalies, and systemic defects, such as the hereditary angiopathy with nephropathy, aneurysms, and muscle cramps syndrome [3,4].

Col IV activates intracellular signaling events to promote cell survival, proliferation, and tumorigenesis [5], and the aberrant expression of *COL4A1* is associated with tumor progression. For example, *COL4A1* overexpression plays a pivotal role in the carcinogenesis and metastasis of various cancers [6,7,8,9,10]. In gastric cancer, *COL4A1* overexpression is related to trastuzumab resistance and tumor recurrence, leading to poor patient outcomes [11,12,13]. Furthermore, in gliomas, collagen genes, including *COL4A1*, are involved in immune infiltration and epithelial–mesenchymal transition [14].

The tumor microenvironment (TME) plays an essential role in the progression and development of treatment resistance in numerous malignancies [15,16,17]. The TME is an ecosystem that surrounds a tumor, consisting of immune cells, stromal fibroblasts, endothelial cells, and non-cellular components, such as the extracellular matrix and soluble factors [15,16,17,18,19]. Macrophages affect tumor cells by releasing cytokines, chemokines, enzymes, arachidonic acid metabolites, and reactive radicals via cell–cell interactions and fluid phase-mediated mechanisms [20]. The macrophages recruited in the TME, namely tumor-associated macrophages (TAMs), are the most abundant immune cells in the TME [21] and play an important role in tumor progression. In response to different stimuli, TAMs can differentiate into two distinct phenotypes, M1 and M2; the M1 phenotype enhances the Th1 response and mediates pro-inflammatory behaviors, whereas the M2 phenotype promotes the Th2 response and displays anti-inflammatory functions associated with tumor progression, invasion, and metastasis, and suppression of T cell immunity [22,23,24]. Therefore, M1 macrophages are generally considered potent effector cells that kill tumor cells and produce various proinflammatory cytokines [25]. In contrast, M2 macrophages promote angiogenesis, tissue remodeling, and repair induced by various signals (such as interleukin (IL)-4, IL-13, glucocorticoids, IL-10, and immunoglobulin complexes/TLR ligands) [23,26]. Immunosuppression is a well-known mechanism of tumor progression that leads to tumor growth and metastasis [27]. Regulatory T cells (Tregs) are potent immune suppressors. Tregs as well as M2 macrophages inhibit the anticancer functions of various effector cells, such as the natural killer (NK), CD8^+^ T, and γδ T cells, thereby inducing metastasis and tumor cell growth in the TME [21,28,29,30,31,32]. Furthermore, stromal cells, other than immune cells in the TME, such as cancer-associated fibroblasts (CAFs) and tumor endothelial cells (TECs), play multifaceted roles as regulators of tumor progression. Thus, CAFs that construct the stroma of the TME support tumor angiogenesis and function as key mediators of immune regulation [33]. Similar to the positive roles of CAFs in tumor progression, TECs serve as major gatekeepers for TMEs infiltrating immune cells and are involved in direct anti-cancer immune responses [34].

The TME represents a complex network of tumor cells that interacts with various cell types. Recently, new technology platforms have shed light on the analysis of the cellular composition of TME at high resolutions and identified a complex landscape of multi-lineage immune and stromal cells, such as CAF and TECs. In this study, using various bioinformatics databases available from public resources, we analyzed the levels of *COL4A1* expression in 33 types of tumor tissues and examined whether elevated *COL4A1* levels are associated with poor prognosis depending on the type of tumor. Furthermore, we investigated the potential role of elevated *COL4A1* expression on the modulation of the immune landscape of TME to assess the prognostic value of *COL4A1* expression as a biomarker, thereby providing novel insights into immunotherapeutic avenues for better treatment of patients with cancer.

## 2. Material and Methods

### 2.1. Analysis of COL4A1 mRNA Expression Levels between Cancer and Normal Tissues

We compared the mRNA expression levels of *COL4A1* between tumor and normal tissues of multiple cancer types using Gene Expression Profiling Interactive Analysis (GEPIA, available at http://gepia.cancer-pku.cn/detail.php; accessed on 26 January 2021) and the Gene Expression Database of Normal and Tumor Tissues 2 (GENT2, available at http://gent2.appex.kr/gent2/; accessed on 26 January 2021). In GEPIA web tool, we compared *COL4A1* expression levels across tumor samples using The Cancer Genome Atlas (TCGA) and Gene Expression database of Normal and Tumor tissues (GTEx) datasets [35]. A Dot blot of *COL4A1* expression profile was retrieved from the Single Gene Analysis module. In GENT2, an integrated database of microarray-based expression datasets, we compared the transcription levels of *COL4A1* between tumors and corresponding normal tissues for multiple types of cancer in the HG-U133_Plus_2 datasets [36,37]. A box plot was obtained by search with “COL4A1” term in Tissue Type option. Significant test result provided as a table was integrated with bar graph by marking significant differences with asterisks.

### 2.2. Survival Analyses of Cancer Patient Groups with High and Low Expression Levels of COL4A1

Survival analyses were performed using two web-based analysis tools: Easy effective survival analysis tool (ESurv, https://easysurv.net/; accessed on 31 October 2021) [38] and R2 Genomics Analysis and Visualization Platform (R2 platform, https://hgserver1.amc.nl/; accessed on 2 February 2022) [39]. ESurv was used to analyze the relationship between *COL4A1* expression levels and the overall survival of patients with the optimal cut-off option in TCGA datasets. The analysis results for pancreatic adenocarcinoma (PAAD), skin cutaneous melanoma (SKCM), stomach adenocarcinoma (STAD), and low-grade glioma (LGG) datasets are shown in the ESurv webpages (PAAD: https://easysurv.net/#/app/result/general/detail/2608; accessed on 31 October 2021, SKCM: https://easysurv.net/#/app/result/general/detail/2611; accessed on 31 October 2021, STAD: https://easysurv.net/#/app/result/general/detail/2610; accessed on 31 October 2021, and LGG: https://easysurv.net/#/app/result/general/detail/2612; accessed on 31 October 2021). The R2 platform was utilized to analyze the correlation between *COL4A1* gene expression levels and patient survival using the optimal cut-off option to split patient groups into microarray-based datasets of tumor glioma (GSE43378), mixed tumor pancreas (GSE28735), tumor melanoma (GSE65904), and tumor gastric (GSE15459). A COX *p*-value less than 0.05 was regarded to be statistically significant.

### 2.3. Analysis of Heterogenic Expression of COL4A1 and Its Association with Infiltrated CAFs and Endothelial Cells

To examine the expression levels of COL4A1 in various tumor-associated cell types in glioma, PAAD, melanoma, and STAD tissues, we employed two single-cell RNA-sequencing data resources: single cell portal (https://singlecell.broadinstitute.org/single_cell; accessed on 30 April 2021) and Tumor Immune Single Cell Hub (TISCH, http://tisch.comp-genomics.org; accessed on 18 March 2022). In single cell portal, we used a specific accessible study, “Study: Melanoma intra-tumor heterogeneity”, in which profiled 4656 single cells isolated from 19 patients with melanoma [40] to check heterogenic *COL4A1* expression in melanoma TME. The gene expression measured by single-cell RNA-sequencing was visualized in various ways, including scatter and violin plots. In TISCH, we retrieved the expression characteristics of heterogenic *COL4A1* in glioma, PAAD, SKCM, and STAD tumors through 4 different studies (Glioma: 17,185 cells isolated from 8 patients using GSE103224 dataset [41], PAAD: 57,443 cells isolated from 35 patients using CRA001160 dataset [42], SKCM: 4645 cells isolated from 19 patients using GSE72056 [40], STAD: 41,554 cells isolated from 13 patients using GSE134520 [43]). Each single-cell expression data was visualized as a scatter plot.

The immune association module in Tumor IMmune Estimation Resource (TIMER) 2.0 (http://timer.cistrome.org/; accessed on 30 September 2021) [44] was used to examine the correlation between *COL4A1* expression levels and infiltration of CAFs and TECs. The correlations of *COL4A1* expression levels with infiltrated CAFs estimated using the EPIC, MCP-COUNTER, and XCELL algorithms and TECs estimated using TIDE, EPIC, MCP-COUNTER, and XCELL were retrieved from TIMER 2.0.

### 2.4. Analysis of Correlation between Tumor Infiltration and COL4A1 Expression

Correlation of immune infiltration with *COL4A1* expression in TCGA datasets was visualized using a gene module through TIMER [45]. Gene module in the TIMER database (available at https://cistrome.shinyapps.io/timer/; accessed on 31 March 2021) was used to analyze the immune cell infiltration in over 10,897 RNA-sequencing samples in 32 different types of cancer from TCGA [46]. The correlations among *COL4A1* expression and tumor purity and tumor-infiltrating immune cell (B cells, CD8^+^ T cells, CD4^+^ T cells, macrophages, neutrophils, and dendritic cells) infiltration was explored for each cancer. Scatterplots were generated after entering the gene symbol, showing partial Spearman’s rho values and statistical significance at the purity correction [45].

### 2.5. Analyses of Correlations among COL4A1 Expression Levels and Immune Cell Marker Gene and Cytokine Expression Levels

Correlations among the expression levels of *COL4A1* and marker genes of immune cells and immune-suppressive cytokines were analyzed using the TIMER and R2 platforms. A list of marker genes for each type of immune cell was used as previously described [47,48]. Spearman′s correlation values for the expression levels of *COL4A1* and each marker gene in PAAD, SKCM, STAD, and LGG in the TCGA database were explored using the Gene_Corr module in the TIMER tool [44,45]. The “correlated 2 gene” module of R2 platform was utilized to examine the correlation between the expression levels of *COL4A1* and marker genes in the microarray-based datasets GSE43378, GSE28735, GSE65904, and GSE15459.

## 3. Results

### 3.1. mRNA Levels of COL4A1 in Various Types of Tumors

**Figure 1:**
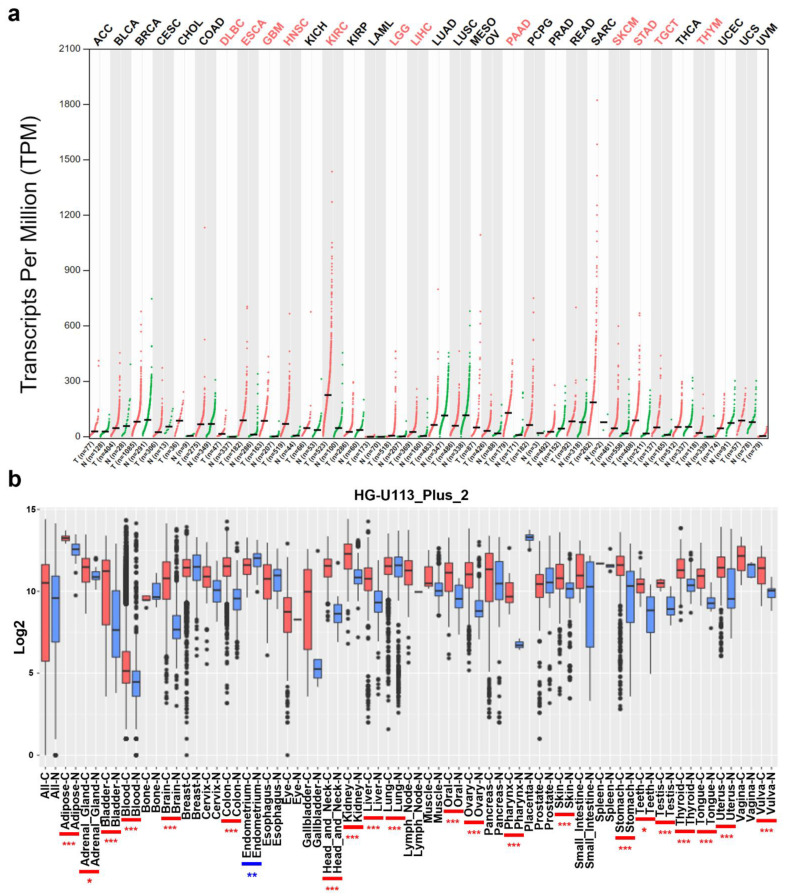
Collagen type IV alpha chain 1 (*COL4A1*) mRNA expression levels in various types of cancer and normal tissues. (**a**) High or low expression levels of *COL4A1* in datasets of 33 types of cancers compared with normal tissues in the Gene expression Profiling Interactive Analysis (GEPIA) database (http://gepia.cancer-pku.cn/detail.php; accessed on 26 January 2021). The graph shown as a dot plot indicates the mRNA expression of each sample. Cancer samples are indicated in red and normal samples in green. Cancer type abbreviations are listed in Table S1. (**b**) Tissue-wide patterns of *COL4A1* expression in 35 human tumors from different tissue origins using the Gene Expression database of Normal and Tumor tissues 2 (GENT2) (http://gent2.appex.kr/gent2/; accessed on 26 January 2021). Boxes show the median, 25th, and 75th percentiles; dots represent the outliers. Significant differences between each tumor tissue and its normal counterpart were indicated by blue or red asterisks (* *p* < 0.05, ** *p* < 0.01, *** *p* < 0.001) for high expression levels in normal tissues or in tumors, respectively.

To compare the mRNA levels of *COL4A1* in various types of tumors with those in normal tissues, we used the GEPIA and GENT2 tools. In GEPIA, the majority of the examined tumors exhibited enhanced *COL4A1* expression, and its expression was significantly upregulated, especially in 12 tumor types, including diffuse large B-cell lymphoma (DLBC), esophageal carcinoma (ESCA), glioblastoma multiforme (GBM), head and neck squamous cell carcinoma (HNSC), kidney renal clear cell carcinoma (KIRC), low-grade glioma (LGG), liver hepatocellular carcinoma (LIHC), pancreatic adenocarcinoma (PAAD), skin cutaneous melanoma (SKCM), stomach adenocarcinoma (STAD), testicular germ cell tumors (TGCT), and thymoma (THYM) (Figure 1a). Additionally, in GENT2 using the platform “HG-U133_Plus_2”, significantly increased *COL4A1* expression was also observed in all the examined tumor tissues from adipose, adrenal gland, bladder, blood, brain, colon, head and neck, kidney, liver, lung, oral, ovary, pharynx, skin, stomach, teeth, testis, thyroid, tongue, uterus, and vulva tumors, except for endometrium tumors in comparison to their normal counterpart tissues (Figure 1b). Collectively, the data from the two analyses showed that *COL4A1* expression was upregulated in most cancer types.

### 3.2. Analysis of Correlation between COL4A1 Expression and Patient Survival

**Figure 2:**
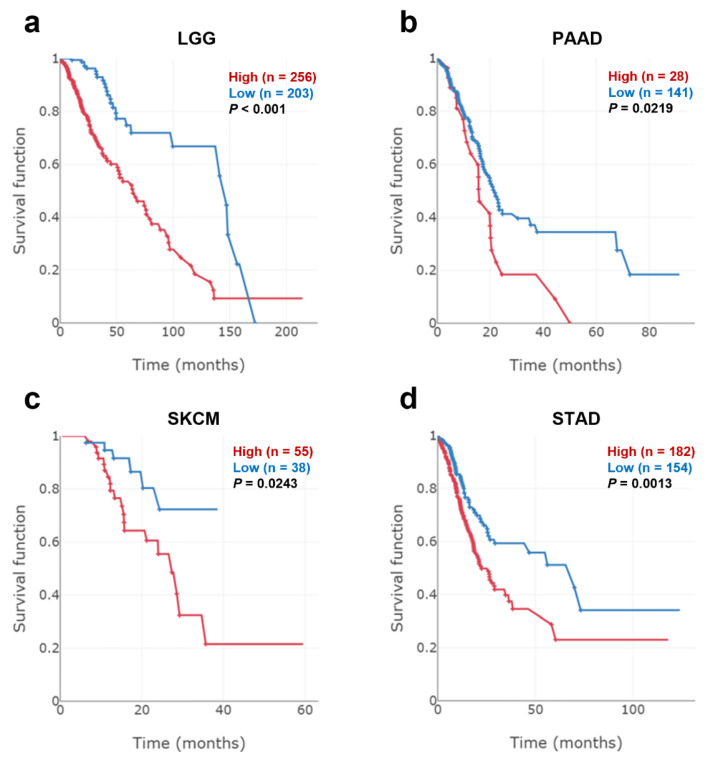
Patient overall survival according to *COL4A1* expression levels in low-grade glioma (LGG), pancreatic adenocarcinoma (PAAD), skin cutaneous melanoma (SKCM), and stomach adenocarcinoma (STAD). Kaplan–Meier survival curves of patient groups with high (red) and low (blue) *COL4A1* expression levels split by optimal cut-off to minimize the *p*-value were retrieved from The Cancer Genome Atlas (TCGA) datasets with ESurv online analysis tool. (**a**) LGG (*n* = 524), (**b**) PAAD (*n* = 169) (**c**) SKCM (*n* = 458), and (**d**) STAD (*n* = 381).

In the previous section, RNA-sequencing data from the TCGA database showed that *COL4A1* was significantly upregulated in 12 different types of tumors. Moreover, tissue-wide RNA microarray data revealed that *COL4A1* was overexpressed in 21 tumor tissues. To identify the cancer types in which increased expression of *COL4A1* was correlated with patient survival, we performed a Kaplan–Meier survival analysis of two patient groups split by optimal cut-off to maximize survival differences in TCGA datasets depending on the expression levels of *COL4A1*. Among the tested types, those with LGG, PAAD, SKCM, STAD, or TGCT displayed a significant correlation between poor prognosis and high *COL4A1* expression (Figure 2 and Figure S1), indicating that the survival probability of patients with LGG, PAAD, SKCM, STAD, and TGCT may be dependent on the level of *COL4A1* expression. Furthermore, in microarray-based datasets of gliomas, pancreatic tumors, melanomas, and gastric cancers, we identified a correlation between the poor survival rates of the four patient groups and high expression levels of *COL4A1*. These results are noteworthy because the four groups are closely related to the tumor cell origins of LGG, PAAD, SKCM, and STAD (Figure S2). Hence, the four tumor types, LGG, PAAD, SKCM, and STAD, were subjected to further analysis in this study, except for TGCT, because no further microarray-based datasets were available for patients with this type of tumor.

### 3.3. High Expression Levels of COL4A1 in Infiltrated CAFs and TECs among Heterogeneous TME Cells

**Figure 3:**
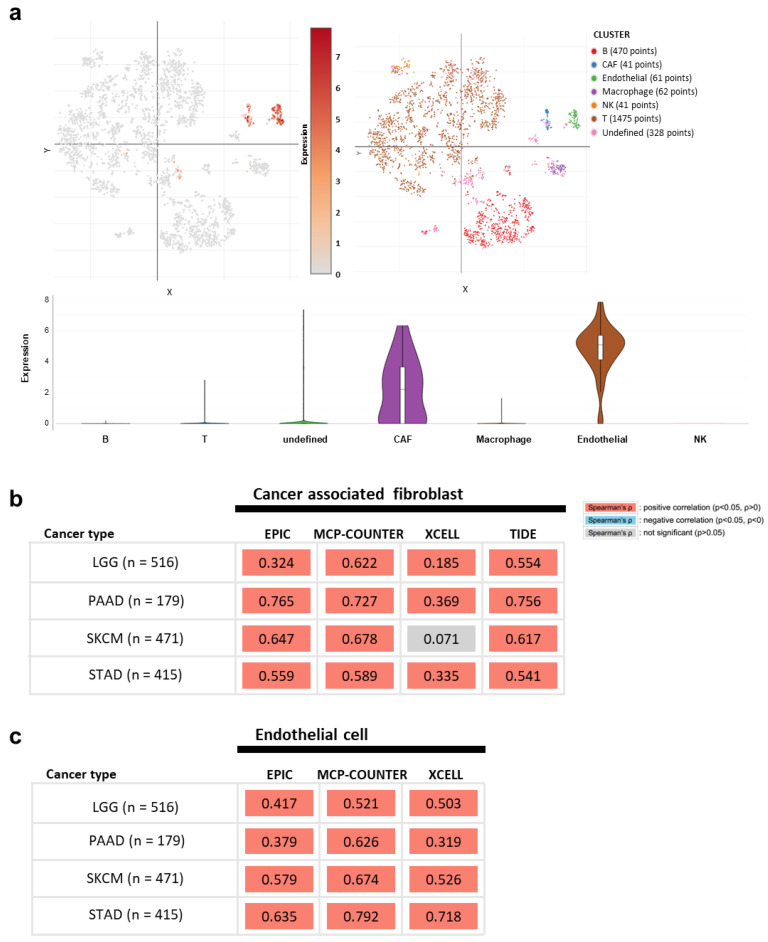
*COL4A1* expression levels in various non-tumoral cells in melanoma TME and correlation of signatures of infiltrated cancer-associated fibroblasts (CAFs) and tumor endothelial cells (TECs) in LGG, PAAD, SKCM, and STAD. (**a**) Analysis of *COL4A1* expression levels in various TME cells using single cell RNA-sequencing data in “Study: Melanoma intra-tumor heterogeneity.” The data are visualized with scatter and violin plots in single cell portal. Correlation values between *COL4A1* expression levels and the signatures of CAFs (**b**) or endothelial cells (**c**) were retrieved using multiple algorithms (EPIC, MC-COUNTER, XCELL, and TIDE for CAFs and EPIC; MC-COUNTER and XCELL for TEC) in TIMER 2.0.

The TME is composed of diverse cell types and secreted factors, and each cell group has divergent gene expression levels that contribute to tumor progression [49,50]. The nature of the TME is closely related to the aggressiveness of malignant cells by orchestrating the immune landscape, subsequently affecting the patient’s prognosis [51,52,53]. As it is not clear which cell types in TME mainly express *COL4A1*, we next analyzed *COL4A1* expression levels in individual cells by “Study: Melanoma intra-tumor heterogeneity” [40] through a Single Cell Portal. Upon analysis, we found that the two major cell types with high *COL4A1* mRNA levels in TME were CAFs and TECs, which are nonmalignant stromal cells (Figure 3a). High expression of COL41 exclusively in CAFs and TECs among various cell types present in TME was also observed in glioma, PAAD, STAD, and SKCM in the analysis using the TISCH database albeit some strong COL41 expression in glioma and STAD malignant cells (Figure S3).

Next, we examined the correlation between high *COL4A1* expression levels and the infiltration of the two types of stromal cells, CAFs and TECs, in LGG, PAAD, SKCM, and STAD as high expression levels of *COL4A1* in these tumor types were correlated with poor patient survival rates in the previously conducted analysis using the TIMER 2.0 tool. The CAF signatures of LGG, PAAD, SKCM, and STAD showed a very strong positive correlation with *COL4A1* expression levels in all four algorithms, except for the result with the XCELL algorithm of SKCM (Figure 3b). In addition, TEC signatures were also highly correlated with *COL4A1* expression levels (Figure 3c). Collectively, these results suggest that the infiltrated levels of CAFs and TECs in the TME are responsible for the increased expression levels of *COL4A1*.

### 3.4. Correlation between COL4A1 Expression and Immune Cell Infiltration

**Figure 4:**
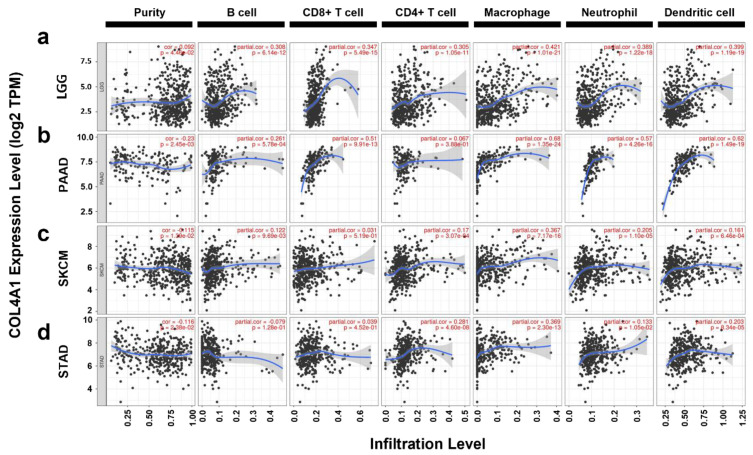
Correlations between immune cell infiltration and *COL4A1* expression levels in LGG, PAAD, SKCM, and STAD. Correlation values between *COL4A1* expression levels and the abundance of various types of infiltrating immune cells in TCGA datasets were retrieved using the TIMER web tool. Correlation analysis between immune infiltration and *COL4A1* expression in (**a**) LGG, (**b**) PAAD, (**c**) SKCM, (**d**) STAD; e.g., 1.19e-19 mean 1.19 × 10^−19^.

To investigate whether the upregulation of *COL4A1* expression induced poor prognosis in LGG, PAAD, SKCM, and STAD by modulating the immune landscape in the TME, we analyzed the association between *COL4A1* expression and infiltration of immune cells into the TME. We utilized the TIMER database using TCGA datasets for correlation studies with the basic types of immune cells frequently seen in TME (29092952). In LGG, the infiltrated levels of B cells (cor. = 0.308, *p* = 6.14 × 10^−12^), CD8^+^ T cells (cor. = 0.347, *p* = 5.49 × 10^−15^), CD4^+^ T cells (cor. = 0.305. *p* = 1.05 × 10^−11^), macrophages (cor. = 0.421, *p* = 1.01 × 10^−21^), neutrophils (cor. = 0.389, *p* = 1.22 × 10^−18^), and dendritic cells (cor. = 0.399, *p* = 1.19 × 10^−19^) were positively correlated with *COL4A1* expression levels (Figure 4a). In PAAD, the infiltrated levels of B cells (cor. = 0.261, *p* = 5.78 × 10^−4^), CD8^+^ T cells (cor. = 0.51, *p* = 9.91 × 10^−13^), macrophages (cor. = 0.68, *p* = 1.35 × 10^−24^), neutrophils (cor. = 0.57, *p* = 4.26 × 10^−16^), and dendritic cells (cor. = 0.62, *p* = 1.49 × 10^−19^) were positively correlated with *COL4A1* expression levels (Figure 4b). However, infiltration of CD4^+^ T cells (cor. = 0.067, *p* = 3.88 × 10^−1^) was not significantly associated with the *COL4A1* expression level in PAAD. In SKCM, the infiltrated levels of B cells (cor. = 0.122, *p* = 9.69 × 10^−3^), CD8^+^ T cells (cor. = 0.031, *p* = 5.19 × 10^−1^), CD4^+^ T cells (cor. = 0.17, *p* = 3.07 × 10^−4^), macrophages (cor. = 0.367, *p* = 7.17 × 10^−16^), neutrophils (cor. = 0.205, *p* = 1.10 × 10^−5^), and dendritic cells (cor. = 0.161, *p* = 6.46 × 10^−4^) were positively correlated with *COL4A1* expression levels (Figure 4c). In STAD, the infiltrated levels of B cells (cor. = –0.079, *p* = 1.28 × 10^−1^) showed a weak negative correlation, while CD8^+^ T cells (cor. = 0.039, *p* = 4.52 × 10^−1^), CD4^+^ T cells (cor. = 0.281, *p* = 4.60 × 10^−8^), macrophages (cor.= 0.369, *p* = 2.30 × 10^−13^), neutrophils (cor.= 0.133, *p* = 1.05 × 10^−2^), and dendritic cells (cor.= 0.203, *p* = 8.34 × 10^−5^) showed positive correlation with *COL4A1* expression levels (Figure 4d). Notably, macrophage signatures among the examined immune cell types displayed the highest correlation with *COL4A1* expression in all four cancer types. In SKCM and STAD, macrophage signatures showed a highly significant correlation with *COL4A1* expression levels, unlike those of CD8^+^ T cells, indicating the potential effect of high *COL4A1* expression on the infiltration of macrophages but not on CD8^+^ T cells as an immune modulator for tumor progression in the TME. In LGG and PAAD cases, despite the relatively high infiltration values of CD8^+^ T cells, their macrophages were even more highly infiltrated, depending on the expression levels of *COL4A1*. These results suggest that the expression levels of *COL4A1* in LGG, PAAD, SKCM, and STAD have a potential influence on the regulation of immune cell recruitment, especially of macrophages, in tumor tissues.

### 3.5. Correlation between the Expression Levels of COL4A1 and Marker Genes Specific to Immune-Suppressive Subtypes

**Figure 5:**
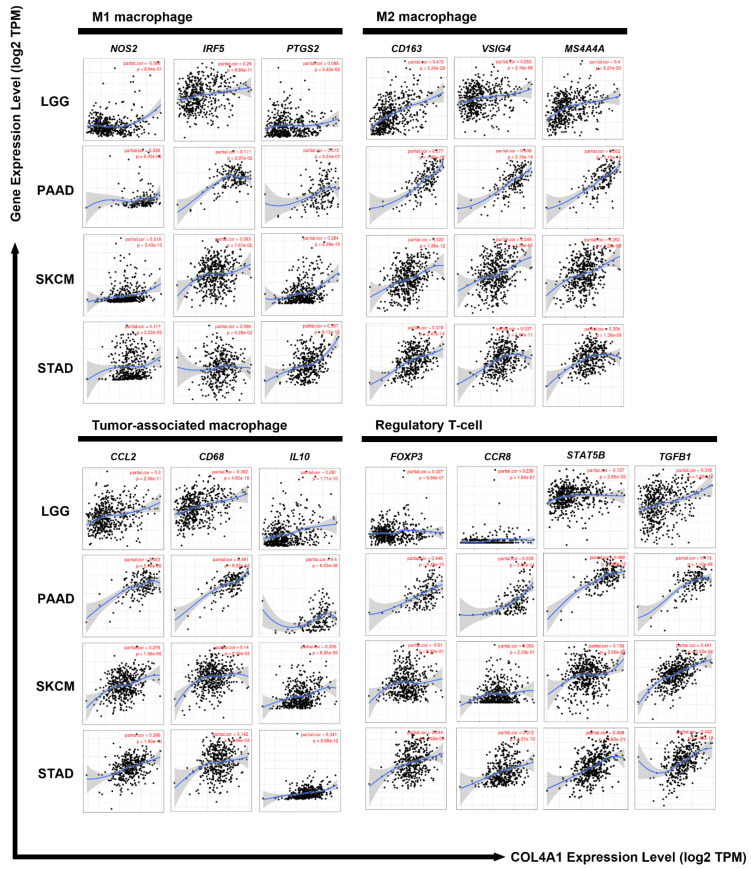
Correlations of *COL4A1* expression levels with the expression levels of M1, M2, TAM, and Treg markers in LGG, PAAD, SKCM, and STAD. M1 macrophages, M2 macrophages, TAM, and Treg cells infiltration levels are portrayed based on *COL4A1* expression with various gene markers using TIMER. Nitric oxide synthase 2 (*NOS2*), interferon regulatory factor 5 (*IRF5*), and prostaglandin-endoperoxide synthase 2 (*PTGS2*) were used as markers for M1 macrophages; *CD163*, V-set and immunoglobulin domain containing 4 (*VSIG4*), and membrane-spanning 4-domains A4A (*MS4A4A*) were used as markers for M2 macrophages; C–C chemokine ligand 2 (*CCL2*), *CD68*, and interleukin-10 (*IL10*) were used as markers for TAM; Forkhead box protein P3 (*FOXP3*), C-C motif chemokine receptor 8 (*CCR8*), signal transducer and activator of transcription 5B (*STAT5B*), and transforming growth factor-β1 (*TGFβ1*) were used as markers for Treg cells. *COL4A1* expression levels were positively correlated with those of various gene markers for M2, TAM, and Treg cells in LGG, PAAD, SKCM, and STAD. Rates of correlation and *p*-values are shown in Table 1, e.g., 1.71e-10 mean 1.71 × 10^−10^.

In the previous section, we identified a high correlation between *COL4A1* expression and macrophage infiltration in LGG, PAAD, SKCM, and STAD. We further examined whether infiltrated macrophages in the four cancer types were tumor-promoting M2-type macrophages as M1-type macrophages are tumor-resistant due to their intrinsic phagocytosis and enhanced antitumor reactions. To identify the type of immune cells influenced by *COL4A1* expression in TME, we further analyzed the correlation between the expression levels of *COL4A1* and the marker genes of various immune cell subtypes, including TAMs that are not a typical type of macrophages and are different from M1 or M2 macrophages, using the TIMER web tool (Table 1 and Table S2). Most of the immune cell marker genes showed a positive correlation with *COL4A1* expression, among which the marker genes specific for M2 macrophages, TAMs, and Treg cells showed a strong positive correlation with *COL4A1* expression levels in LGG, PAAD, SKCM, and STAD (Table 1 and Figure 5). Although some *COL4A1*-expressing cases correlated with specific marker genes for cells with anticancer effects, such as CD8^+^ T cells, M1 macrophages, Th1 cells, and neutrophils, TAM and M2 markers were much more strongly correlated than M1 markers, as shown in Table 1. These data indicate that *COL4A1* expression is exclusively correlated with the marker genes of TAMs and M2 macrophages. Furthermore, we performed additional expression correlation analyses of *COL4A1* with more M1 marker genes (*IL12b* and *CXCL11*) and M2 marker genes (*STAT6*, *IL6*, and *CD206)* using TIMER. *IL12b* is a pro-M1 gene to regulate macrophage activation and polarization [54]. *CXCL11* is a chemokine highly expressed in M1 macrophages, which recruits activated T cells [55]. STAT6 is a well-known driver to polarize macrophages to M2 [56]. IL6 is an M2 macrophage-secreted cytokine that facilitates metastatic activity in cancer cells [57,58]. *CD206* is commonly expressed on M2 macrophages [59]. Upon examining their expression correlation, we revealed that the M2-related genes have a stronger positive correlation with *COL4A1* than the M1-related genes (Figure S4). Treg markers also showed a strong correlation with *COL4A1* expression levels in all four types of tumors, with some variations. In PAAD and STAD cases, a significant positive correlation with all four Treg markers was detected, but the expression levels of forkhead box protein P3 (*FOXP3*) and C-C motif chemokine receptor 8 (*CCR8*) were not significantly correlated with *COL4A1* expression levels in LGG, SKCM, and LGG (Table 1 and Figure 5). *COL4A1* expression was also significantly correlated with the expression levels of T cell exhaustion marker genes such as *PDCD1*, *CTLA4*, *LAG3*, and *HAVCR2* in all the four tumor types with only two exceptions (*CTLA4* in SKCM and *LAG3* in STAD) (Table S2).

Furthermore, to confirm the results with LGG, PAAD, SKCM, and STAD, we examined whether *COL4A1* expression levels were correlated with those of immune marker genes in gliomas, pancreatic tumors, melanomas, and gastric cancers using microarray-based datasets as the *COL4A1* expression predicted poor overall survival in patients with these tumors, as shown in Figure S2. Similar to the TIMER analysis of TCGA datasets, *COL4A1* expression levels were highly correlated with the expression levels of marker genes for TAMs and M2 macrophages (Table S3). In particular, the expression levels of all three marker genes of M2 macrophages were strongly correlated with *COL4A1* expression levels. However, the overall correlation with other immune cells analyzed using microarray-based datasets was less apparent than that using TCGA datasets. Together, these analyses revealed that *COL4A1* expression is highly associated with the infiltration of immune-suppressing cells, such as M2 macrophages, TAMs, and Tregs, suggesting the potential role of high *COL4A1* expression in poor prognosis of patients via immune modulation of the TME, at least in the tumor types analyzed in this study.

### 3.6. Correlation between the Expression Levels of COL4A1 and Immunosuppressive Cytokines

**Figure 6:**
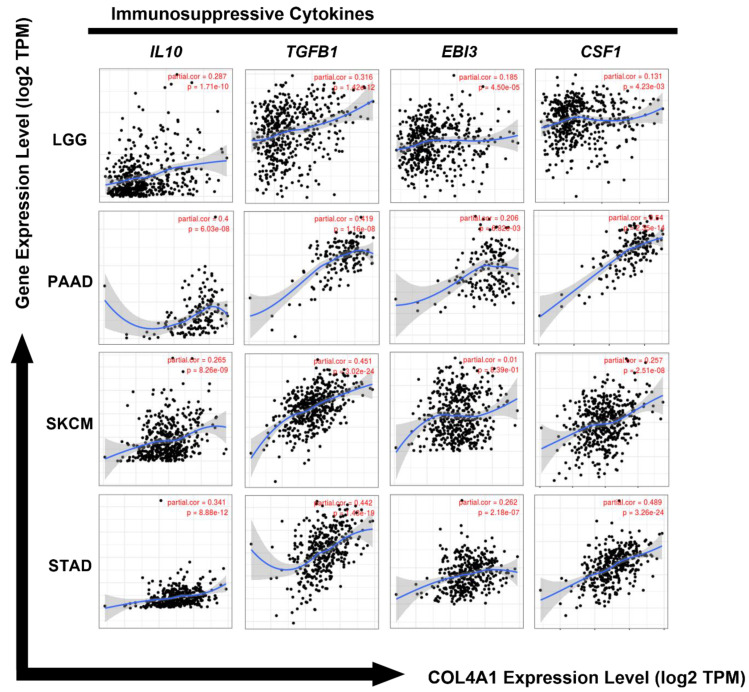
Correlation of *COL4A1* expression levels with the expression levels of immune-suppressive cytokines (IL-10, TGFβ1, Epstein–Barr virus induced 3 (EBI3), and colony-stimulating factor 1 (CSF1)). Correlation analyses among expression levels of *COL4A1* and four cytokine gene markers are depicted using TIMER. Significant positive correlations between the expression levels of *COL4A1* and the four cytokines, IL-10, CSF1, TGFβ1, IL-10, and EBI3, were observed in LGG, PAAD, SKCM, and STAD, e.g., 3.26e-24 mean 3.26 × 10^−24^.

As shown in Figure 5 and Table 1, there was a strong positive correlation between *COL4A1* expression levels and the marker-specific expression levels of Treg cells, M2 macrophages, and TAMs, indicating the potential role of *COL4A1* expression in the infiltration of immune-suppressing cells. To investigate whether the expression levels of cytokine markers derived from these immunosuppressive cells were also increased by *COL4A1* expression, we analyzed the expression levels of cytokine genes (*IL10*, transforming growth factor (*TGF*)-*β1*, Epstein–Barr virus induced 3 (*EBI3*), and colony-stimulating factor 1 (*CSF1*)) in LGG, PAAD, SKAM, and STAD using the TIMER database. IL-10 promotes monocyte differentiation towards an M2 phenotype macrophage and reinforces tumor characteristics, including cell proliferation and metastasis, by exerting immunosuppressive effects [60,61]. TGFβ1 promotes epithelial–mesenchymal transition and is associated with increased tumor cell motility and invasion [62]. EBI3, composed of IL-35, inhibits the differentiation and functions of Th1 and Th17 cells by promoting the expansion of Tregs and production of IL-10 [63,64,65]. CSF1 increases the levels of immune-suppressing M2 macrophages by regulating macrophage proliferation and differentiation [66]. As shown in Figure 6, the expression levels of *COL4A1* were positively correlated with the gene expression levels of these immunosuppressive cytokines in all four analyzed tumor types, LGG (*IL10*: cor. = 0.287, *p* = 1.71 × 10^−10^; *TGFβ1*: cor. = 0.316, *p* = 1.42 × 10^−12^; *EBI3*: cor. = 0.185, *p* = 4.50 × 10^−5^; *CSF1*: cor. = 0.131, *p* = 4.23 × 10^−3^), PAAD (*IL10*: cor. = 0.4, *p* = 6.03 × 10^−8^; *TGFβ1*: cor. = 0.419, *p* = 1.16 × 10^−8^; *EBI3*: cor. = 0.206, *p* = 6.82 × 10^−3^; *CSF1*: cor. = 0.54, *p* = 2.35 × 10^−14^), SKCM (*IL10*: cor. = 0.265, *p* = 8.26 × 10^−9^; *TGFβ1*: cor. = 0.451, *p* = 3.02 × 10^−24^; *EBI3*: cor. = 0.01, *p* = 8.39 × 10^−1^; *CSF1*: cor. = 0.257, *p* = 2.51 × 10^−8^), and STAD (*IL10*: cor. = 0.341, *p* = 8.88 × 10^−12^; *TGFβ1*: cor. = 0.442, *p* = 1.43 × 10^−19^; *EBI3*: cor. = 0.262, *p* = 2.18 × 10^−7^; *CSF1*: cor. = 0.489, *p* = 3.26 × 10^−24^). The levels of these immunosuppressive cytokines are frequently elevated in the TME, and they play a crucial role in predicting the poor prognosis of affected patients [67,68,69,70]. Collectively, our data showed that high expression levels of *COL4A1* are associated with pro-tumor effects via upregulation of the expression levels of immunosuppressive cytokines, thereby potentially affecting the poor prognosis of patients with LGG, PAAD, SKCM, and STAD.

TGF-β is a well-known EMT driver, high signaling of which promotes metastatic and invasive growth of tumor cells [71]. Since the COL4A1 expression level was positively correlated with that of *TGF-β1* (Figure 6), we further examined the correlation of *COL4A1* expression with epithelial-mesenchymal transition (EMT)-related genes by retrieving epithelial signature genes (*CDH1*, *DSP*, *OCLN*, and *DSG3*) and mesenchymal signature genes (*CDH2*, *VIM*, *FN1*, *TWIST1*, and *ACTA2*) using TIMER. Among the examined genes, the mesenchymal signatures have a stronger positive correlation with COL4A1 expression with a high statistical significance, compared to epithelial signatures (Figure S5).

## 4. Discussion

Despite the improvements in patient survival in the last 30 years due to the development of novel innovative therapies, including targeted therapy, cancer still is one of the most serious diseases threatening human health worldwide. In 2017, 24.5 million cancer cases were reported worldwide, with 9.6 million deaths [72]. Elucidation of novel molecular targets and/or markers is necessary to develop novel targeted therapies. COL4A1 is generally located in the basement membrane and is thought to be a barrier to tumor invasion. However, recent studies have revealed that the expression levels of *COL4A1* in the TME have a positive relationship with drug resistance and tumor recurrence or progression in certain types of cancer [7,9,13,73]. In this study, we demonstrated that COL4A1 mRNA expression levels are upregulated in various cancer types, and high expression of COL4A1 correlates with poor prognosis in at least four tumor types: LGG, PAAD, SKCM, and STAD. LGGs account for 10–20% of all primary brain tumors and show slower growth than their high-grade counterparts [74,75]. However, any LGG can become life-threatening as the growing tumor may damage vital areas of the brain. PAAD accounts for approximately 85% of all pancreatic cancer cases and has a very poor prognosis, with only 24% of all patients surviving for one year and 6% surviving for five years or more after diagnosis. [76,77]. SKCM is a type of malignant skin cancer that originates from melanocytes and shows an increasing incidence [78,79]. STAD is the third leading cause of cancer-related deaths worldwide and the fifth most diagnosed cancer according to statistics from GLOBOCAN 2018 [80]. Therefore, our study suggests that modulation of COL4A1 expression may be a potential target for the development of novel therapeutic strategies for the treatment of patients with these tumors that show poor prognosis.

Interestingly, *COL4A1* expression levels were significantly upregulated in most cancer types in RNA-sequencing-based TCGA datasets and the integrated microarray-based cancer-expression database GENT (Figure 1), implying the potential correlation of *COL4A1* expression with tumorigenesis or tumor progression. Furthermore, we found statistically significant differences in the overall survival of patients between the high and low *COL4A1* expression level groups in LGG, PAAD, SKCM, and STAD TCGA and microarray-based datasets of corresponding cancer types, suggesting that high *COL4A1* expression is a possible marker for predicting the poor prognosis of patients (Figure 2). Unlike the other three cancer types, the survival rates of the LGG patient group with low *COL4A1* expression declined significantly from approximately 150 months, eventually disappearing after approximately 170 months (Figure 2a). All patients with LGGs eventually progress to high-grade gliomas and die [81]. As the pathological significance of *COL4A1* expression in the progression of LGG remains unknown, it is worth examining the role of *COL4A1* overexpression at the late stage of disease progression after 150 months. Despite some discrepancies in late-stage LGG progression, the association of *COL4A1* overexpression with patient overall survival suggests the existence of a common tumor-promoting mechanism related to *COL4A1* expression, at least in the four tumors analyzed.

The predominant expression of *COL4A1* in CAFs has been previously reported in pancreatic ductal adenocarcinoma [82]. Together with this report, single-cell sequencing data showing that *COL4A1* mRNA is expressed mainly in CAFs and TECs (Figure 3a and Figure S3), is noteworthy because recent studies have proved that CAFs directly and/or indirectly influence immunosuppression in the TME [83,84,85]. CAF-educated myeloid cells are transformed into pro-tumor macrophages, leading to the suppression of T cell proliferation by upregulating TGFB1 expression and IL10 production [86]. This might be the reason for the strong correlations among *COL4A1*, *TGFB1*, and *IL10* expression levels. CAF markers are positively correlated with FoxP3^+^ cells and negatively correlated with CD8^+^ T cells, which may lead to poor prognosis [87,88]. In our study, *COL4A1* expression levels were positively correlated with those of most Treg markers, except FoxP3, in LGG and SKCM (Table 1 and Figure 5). The cytotoxic activity of NK cells is also affected by CAFs [89]. In this context, *COL4A1* is potentially associated with CAF by modulating the immunosuppressive TME toward pro-tumor effects, resulting in worse prognostic outcomes.

We also found that *COL4A1* mRNA is highly expressed in TECs and that high *COL4A1* expression correlates with the level of endothelial infiltration in the tumor mass. Endothelial cells constitute blood vessels, supply metabolic substrates to tumors, and secrete angiocrine factors to facilitate the metastasis of angiogenesis-dependent cancers [90,91,92]. Endothelial cells in the tumor vasculature actively induce the escape of malignant cells and suppress the effects of T cells [93]. COL4A1 is a structural component of the basement membrane in the blood vessels [94]. As angiogenesis enhances tumor progression, it is a representative prognostic factor that worsens patient survival [95]. In addition, tumor-associated blood vessels promote the inflow of immune cells into the tumors [96]. Therefore, our finding showing a positive correlation between *COL4A1* expression levels and TEC signatures suggests that *COL4A1* may be involved in angiogenesis, which is a critical step in tumor progression.

T cells and macrophages are regulators of tumor immunity. Among them, Treg, M2 macrophages, and TAMs suppress immunity and stimulate tumor progression [97,98,99]. In our study, *COL4A1* expression levels showed strong correlations with the levels of infiltrated Tregs, TAMs, and M2 macrophages as well as immunosuppressive cytokine expression levels (Figure 4, Figure 5 and Figure 6, Figure S4 and Table 1). High infiltration of Tregs, TAMs, and M2 macrophages is associated with poor prognosis in patients with cancer [100,101,102]. Interestingly, *COL4A1* expression is highly correlated with T cell exhaustion makers including *PDCD1*, *CTLA4*, *LAG3*, and *HAVCR2*, which are immune checkpoint targets of immunotherapy. However, the immunological roles of *COL4A1* and its relevance to immune cells in tumors have not yet been explored. Therefore, this study indicates the potential relationship between *COL4A1*, which is overexpressed in the TME, and cancer immunity.

We found a higher correlation of *COL4A1* expression with the expression levels of mesenchymal signature genes than epithelial signature genes (Figure S5). However, this correlation cannot imply that elevated expression of COL4A1 enhances poor prognosis by promoting the epithelial-to-mesenchymal transition of tumor cells because these results may reflect altered expression patterns of COL4A1 in mesenchymal origin cells like fibroblasts and endothelial cells in TME, rather in the malignant cells. Indeed, the single-cell RNA-sequencing analysis shown in Figure S3 demonstrated that the COL4A1 is dominantly expressed in fibroblasts and endothelial cells, but not in the malignant cells of LGG, PAAD, and SKCM TME.

Expression of *COL4A2* is highly correlated with that of *COL4A1* in all types of cancers in TIMER2 analysis and exclusively expressed in stromal cells like *COL4A1* in single-cell sequencing data (data not shown). However, other members of Col IV did not show a redundant expression pattern with *COL4A1* (data not shown). The *COL4A1* and the *COL4A2* genes are located adjacently in the head-to-head position and share a bidirectional promoter in chromosome 13 [103]. The coordinated expression driven by the shared promoter could provide proper amounts of COL4A1 and COL4A2 to form a functional 1-1-2 triplex helix. Therefore, it would be intriguing to examine that our results with *COL4A1* may be applied equally to *COL4A2*.

Although our data clearly show the relevance of *COL4A1* expression and immunosuppressive TME in LGG, PAAD, SKCM, and STAD, there are several limitations to our study. First, our analysis used only publicly available transcriptional data from the TCGA database, microarray-based datasets, and single-cell sequencing databases. Second, our analysis was based only on the mRNA expression levels of *COL4A1*; therefore, further studies should be conducted to elucidate the underlying molecular mechanisms. The noncollagenous 1 domain of COL4A1 binds to α1β1 integrin and subsequently regulates the FAK/c-Raf/MEK/ERK1/2/p38 MAPK in endothelial cells [104]. However, although this study revealed that *COL4A1* expression affects macrophage differentiation in TME, the downstream nor COL4A1 receptors of the myeloid cells have not been reported and remain to be explored. Moreover, to confirm the role of *COL4A1* expression in immune infiltration into the TME during tumor growth, further in vitro and in vivo studies should be conducted in the future.

Overall, the results of our study suggest that *COL4A1* overexpression in stromal cells may function as a potential regulator of the tumor-supporting TME composition associated with the poor prognosis of patients with LGG, PAAD, SKCM, and STAD.

## Figures and Tables

**Table 1:** Correlation analyses among the expression levels of *COL4A1* and related marker genes of immune cells in the tumor microenvironment (TME) of four tumor types using the Tumor Immune Estimation Resource (TIMER) tool.

Description	Gene Markers	LGG				PAAD				SKCM				STAD			
		None		Purity		None		Purity		None		Purity		None		Purity	
		Cor	*p*	Cor	*p*	Cor	*p*	Cor	*p*	Cor	*p*	Cor	*p*	Cor	*p*	Cor	*p*
CD8^+^ T cells	*CD8A*	0.149	***	0.203	***	0.367	***	0.303	***	−0.001	0.984	−0.088	0.059	0.133	**	0.109	*
	*CD8B*	0.069	0.117	0.11	*	0.297	***	0.227	**	−0.025	0.583	−0.126	**	0.044	0.37	0.031	0.55
TAMs	*CCL2*	0.282	***	0.3	***	0.344	***	0.303	***	0.302	***	0.278	***	0.385	***	0.366	***
	*CD68*	0.35	***	0.382	***	0.533	***	0.491	***	0.179	***	0.14	**	0.171	***	0.142	**
	*IL10*	0.28	***	0.287	***	0.442	***	0.4	***	0.283	***	0.265	***	0.349	***	0.341	***
M1 macrophages	*NOS2*	−0.039	0.377	−0.006	0.894	0.315	***	0.338	***	0.326	***	0.318	***	0.105	*	0.117	*
	*IRF5*	0.235	***	0.29	***	0.2	**	0.177	*	0.129	**	0.083	0.077	0.107	*	0.096	0.063
	*PTGS2*	0.079	0.073	0.088	0.054	0.354	***	0.373	***	0.3	***	0.284	***	0.369	***	0.357	***
M2 macrophages	*CD163*	0.486	***	0.473	***	0.631	***	0.577	***	0.332	***	0.322	***	0.4	***	0.378	***
	*VSIG4*	0.232	***	0.253	***	0.595	***	0.538	***	0.263	***	0.244	***	0.341	***	0.337	***
	*MS4A4A*	0.393	***	0.4	***	0.614	***	0.562	***	0.28	***	0.262	***	0.319	***	0.305	***
Neutrophils	*CEACAM8*	−0.003	0.944	−0.022	0.628	0.171	*	0.115	0.133	0.075	0.102	0.086	0.066	0.064	0.191	0.084	0.103
	*ITGAM*	0.181	***	0.226	***	0.513	***	0.444	***	0.263	***	0.237	***	0.362	***	0.35	***
	*CCR7*	0.368	***	0.395	***	0.274	***	0.219	**	0.045	0.327	−0.04	0.398	0.27	***	0.261	***
Th1	*TBX21*	0.439	***	0.434	***	0.237	**	0.183	*	0.034	0.465	−0.054	0.247	0.187	***	0.188	***
	*STAT4*	−0.076	0.086	−0.036	0.43	0.274	***	0.27	***	0.122	**	0.071	0.13	0.246	***	0.233	***
	*STAT1*	0.507	***	0.515	***	0.518	***	0.472	***	0.062	0.177	0.017	0.721	0.129	**	0.112	*
	*IFNG*	0.206	***	0.224	***	0.264	***	0.216	**	−0.025	0.585	−0.114	*	−0.01	0.837	−0.014	0.781
	*TNF*	−0.031	0.486	−0.032	0.487	0.263	***	0.233	**	0.046	0.323	−0.028	0.555	0.163	***	0.146	**
Treg	*FOXP3*	0.004	0.927	0.027	0.556	0.495	***	0.448	***	0.066	0.152	−0.01	0.839	0.256	***	0.244	***
	*CCR8*	0.22	***	0.236	***	0.578	***	0.539	***	0.112	*	0.059	0.209	0.324	***	0.312	***
	*STAT5B*	0.166	***	0.137	**	0.443	***	0.499	***	0.13	**	0.136	**	0.469	***	0.458	***
	*TGFB1*	0.292	***	0.316	***	0.441	***	0.419	***	0.454	***	0.451	***	0.465	***	0.442	***

TAM, tumor-associated macrophage; Th, T helper cell; Tfh, follicular helper T cell; Treg, regulatory T cell; Cor, R value of Spearman’s correlation; None, correlation without adjustment. Purity, correlation adjusted for purity. * *p* < 0.05, ** *p* < 0.01, *** *p* < 0.001.

## Data Availability

The data presented in this study are available upon request from the corresponding authors.

## Supplementary Materials

The following are available online at https://www.mdpi.com/article/10.3390/jpm12040534/s1, Figure S1: Overall patient survival according to the expression levels of collagen type IV alpha chain 1 (*COL4A1*) in diffuse large B-cell lymphoma (DLBC), esophageal carcinoma (ESCA), glioblastoma multiforme (GBM), head and neck squamous cell carcinoma (HNSC), kidney renal clear cell carcinoma (KIRC), liver hepatocellular carcinoma (LIHC), testicular germ cell tumors (TGCT), and thymoma (THYM); Figure S2: Kaplan–Meier survival curves of patient groups with high and low *COL4A1* expression levels retrieved from the R2 platform; Figure S3: Single cell RNA-sequencing analysis of COL4A1 gene in TME cells of LGG, PAAD, SKCM, and STAD; Figure S4: The correlation between polarized macrophage-related genes with COL4A1 expression in LGG, PAAD, SKCM, and STAD; Figure S5: The correlation between EMT signature genes with COL4A1 expression in LGG, PAAD, SKCM, and STAD; Table S1: List of The Cancer Genome Atlas (TCGA) abbreviations for different types of cancer; Table S2: Correlation analyses among the expression levels of collagen type IV alpha chain 1 (*COL4A1*) and related marker genes of T, B, monocytes, natural killer (NK), dendritic, Th2, Tfh, Th17, and exhausted T cells in low-grade glioma (LGG), pancreatic adenocarcinoma (PAAD), skin cutaneous melanoma (SKCM), and stomach adenocarcinoma (STAD)-TCGA datasets using the Tumor Immune Estimation Resource (TIMER) web tool; Table S3: Correlation analyses among the expression levels of *COL4A1* and related marker genes of the tumor microenvironment (TME) immune cells of four tumor groups from different origins using the R2 tool.
