# Supplementary material for: Association of Elevated Expression Levels of COL4A1 in Stromal Cells with an Immunosuppressive Tumor Microenvironment in Low-Grade Glioma, Pancreatic Adenocarcinoma, Skin Cutaneous Melanoma, and Stomach Adenocarcinoma
Source: J Pers Med. 2022 Mar 28;12(4):534. doi: 10.3390/jpm12040534 (PMC9029283; doi:10.3390/jpm12040534)
Supplement: Supplementary file 1 [file jpm-12-00534-s001.zip › jpm-1634006-supplementary_corrected.pdf]

Association of Elevated Expression Levels of *COL4A1* in Stromal Cells with Immunosuppressive Tumor Microenvironment in Low-Grade Glioma, Pancreatic Adenocarcinoma, Skin Cutaneous Melanoma, and Stomach Adenocarcinoma

Hyo Jae Shin [1], Minchan Gil [2,*] and Im-Soon Lee [3,*]

[1] Department of Biological Sciences, Konkuk University, 120 Neungdong-ro, Gwangjin-gu, Seoul 05029, Republic of Korea; Shinhyojae0606@gmail.com.
[2] Department of Stem Cell and Regenerative Biotechnology, Konkuk University, 120 Neungdong-ro, Gwangjin-gu, Seoul 05029, Republic of Korea; e-mail@e-mail.com
[3] Department of Biological Sciences, Konkuk University, 120 Neungdong-ro, Gwangjin-gu, Seoul 05029, Republic of Korea; islee@konkuk.ac.kr.
* Correspondence: islee@konkuk.ac.kr

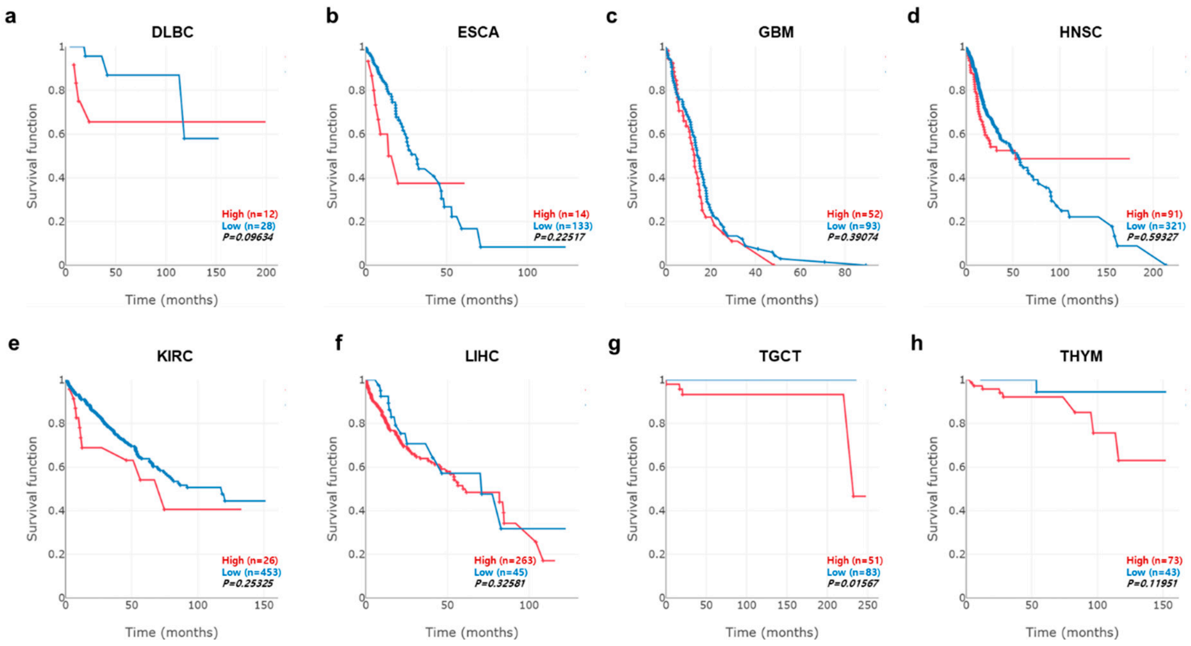

Figure S1. Overall patient survival according to the expression levels of collagen type IV alpha1 chain 1 (*COL4A1*) in diffuse large B-cell lymphoma (DLBC), esophageal carcinoma (ESCA), glioblastoma multiforme (GBM), head and neck squamous cell carcinoma (HNSC), kidney renal clear cell carcinoma (KIRC), liver hepatocellular carcinoma (LIHC), testicular germ cell tumors (TGCT), and thymoma (THYM). Kaplan–Meier survival curves of patient groups with high (red) and low (blue) *COL4A1* expression levels split by optimal cut-off to minimize the *p-value* were retrieved from The Cancer Genome Atlas (TCGA) datasets using the ESurv online analysis tool. (a) DLBC (n = 40), (b) ESCA (n = 147), (c) GBM (n = 145), and (d) HNSC (n = 412), (e) KIRC (n = 479), (f) LIHC (n = 308), (g) TGCA (n = 134), (h) THYM (n = 116).

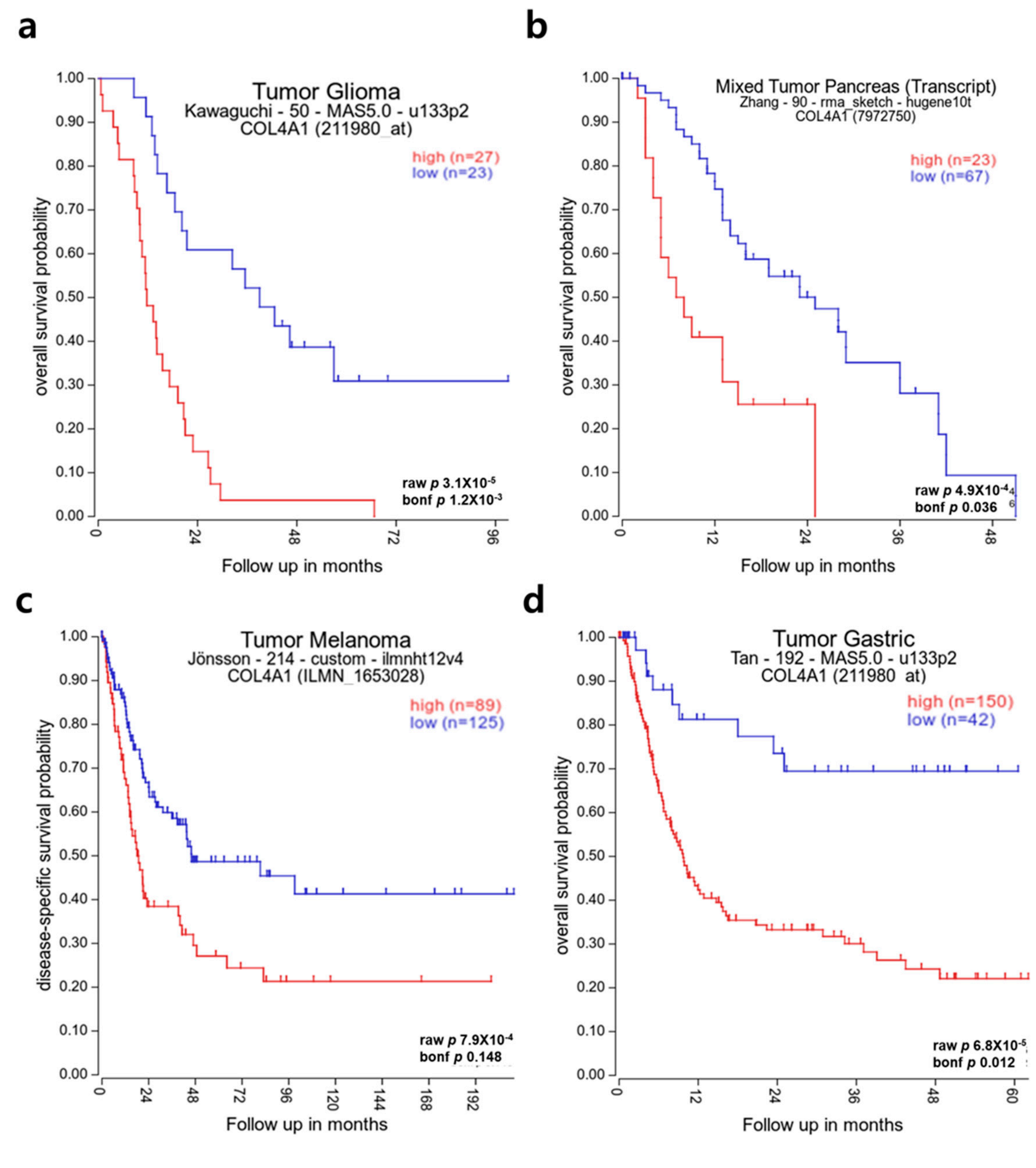

Figure S2. Kaplan–Meier survival curves of patient groups with high and low *COL4A1* expression levels retrieved from the R2 platform. Kaplan–Meier survival curves of the patient groups with high (red) or low (blue) *COL4A1* expression levels were retrieved from the R2 platform. Patients were divided into two groups based on *COL4A1* expression levels to maximize the survival difference. The analyzed datasets were as follows: (a) tumor glioma (GSE43378, n = 50), (b) mixed tumor pancreas (GSE28735, n = 90), (c) tumor melanoma (GSE65904, n = 214), and (d) tumor gastric (GSE15459, n = 192).

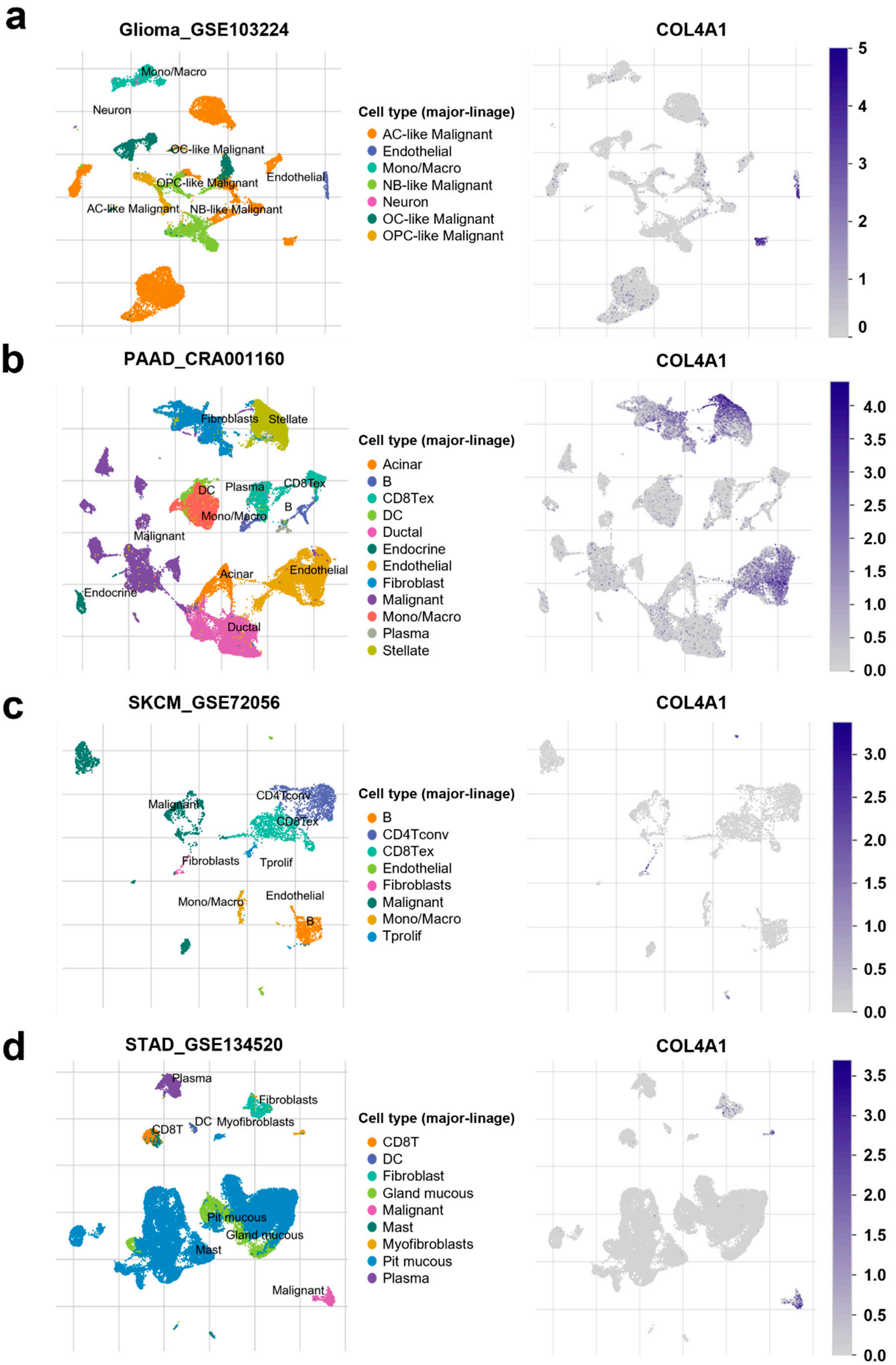

a
Glioma_GSE103224
Mono/Macro
Neuron
OC-like Malignant
OPC-like Malignant
Endothelial
AC-like Malignant
NB-like Malignant
Cell type (major-linage)
AC-like Malignant
Endothelial
Mono/Macro
NB-like Malignant
Neuron
OC-like Malignant
OPC-like Malignant
COL4A1
5
4
3
2
1
0
b
PAAD_CRA001160
Fibroblasts
Stellate
DC
Plasma
CD8Tex
B
Mono/Macro
Malignant
Acinar
Endothelial
Endocrine
Ductal
Cell type (major-linage)
Acinar
B
CD8Tex
DC
Ductal
Endocrine
Endothelial
Fibroblast
Malignant
Mono/Macro
Plasma
Stellate
COL4A1
4.0
3.5
3.0
2.5
2.0
1.5
1.0
0.5
0.0
c
SKCM_GSE72056
Malignant
CD4Tconv
CD8Tex
Fibroblasts
Tprolif
Mono/Macro
Endothelial
B
Cell type (major-linage)
B
CD4Tconv
CD8Tex
Endothelial
Fibroblasts
Malignant
Mono/Macro
Tprolif
COL4A1
3.0
2.5
2.0
1.5
1.0
0.5
0.0
d
STAD_GSE134520
Plasma
Fibroblasts
CD8T
DC
Myofibroblasts
Pit mucous
Gland mucous
Mast
Malignant
Cell type (major-linage)
CD8T
DC
Fibroblast
Gland mucous
Malignant
Mast
Myofibroblasts
Pit mucous
Plasma
COL4A1
3.5
3.0
2.5
2.0
1.5
1.0
0.5
0.0

Figure S3. Single cell RNA-sequencing analysis of the *COL4A1* gene in TME cells of LGG, PAAD, SKCM, and STAD. Single cell RNA-sequencing plots of individual type of cancers with the expression of COL4A1 were retrieved from TISCH dataset browser. Analyzed cancer types were: (a) Glioma (GSE103224; 8 patients, 17185 cells), (b) PAAD (CRA001160; 35 patients, 57443 cells), (c) SKCM (GSE72056; 19 patients, 4645 cells), and (d) STAD (GSE134520; 13 patients, 41554 cells).

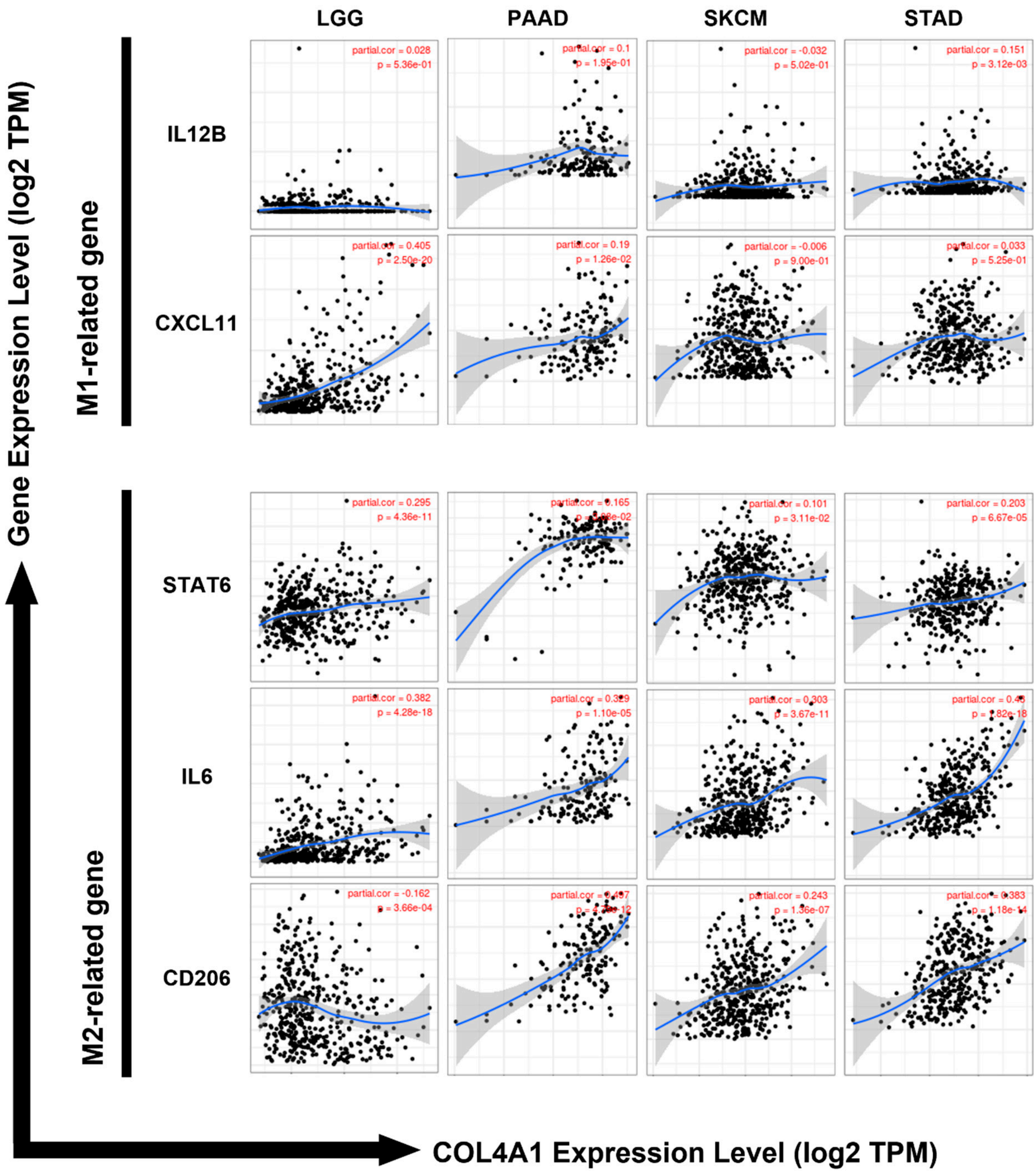

Figure S4. The correlation of *COL4A1* expression with the expression levels of various polarized macrophage-related genes in LGG, PAAD, SKCM, and STAD. Correlation analysis of individual type of cancers were retrieved from TIMER tool. Interleukin-12 subunit beta (*IL12B*) and C-X-C motif chemokine ligand 11 (*CXCL11*) were used as M1-related genes; Signal transducer and activator of transcription 6 (*STAT6*), interleukin-6 (*IL6*), and cluster of differentiation 206 (*CD206*) were used as M2-related genes.

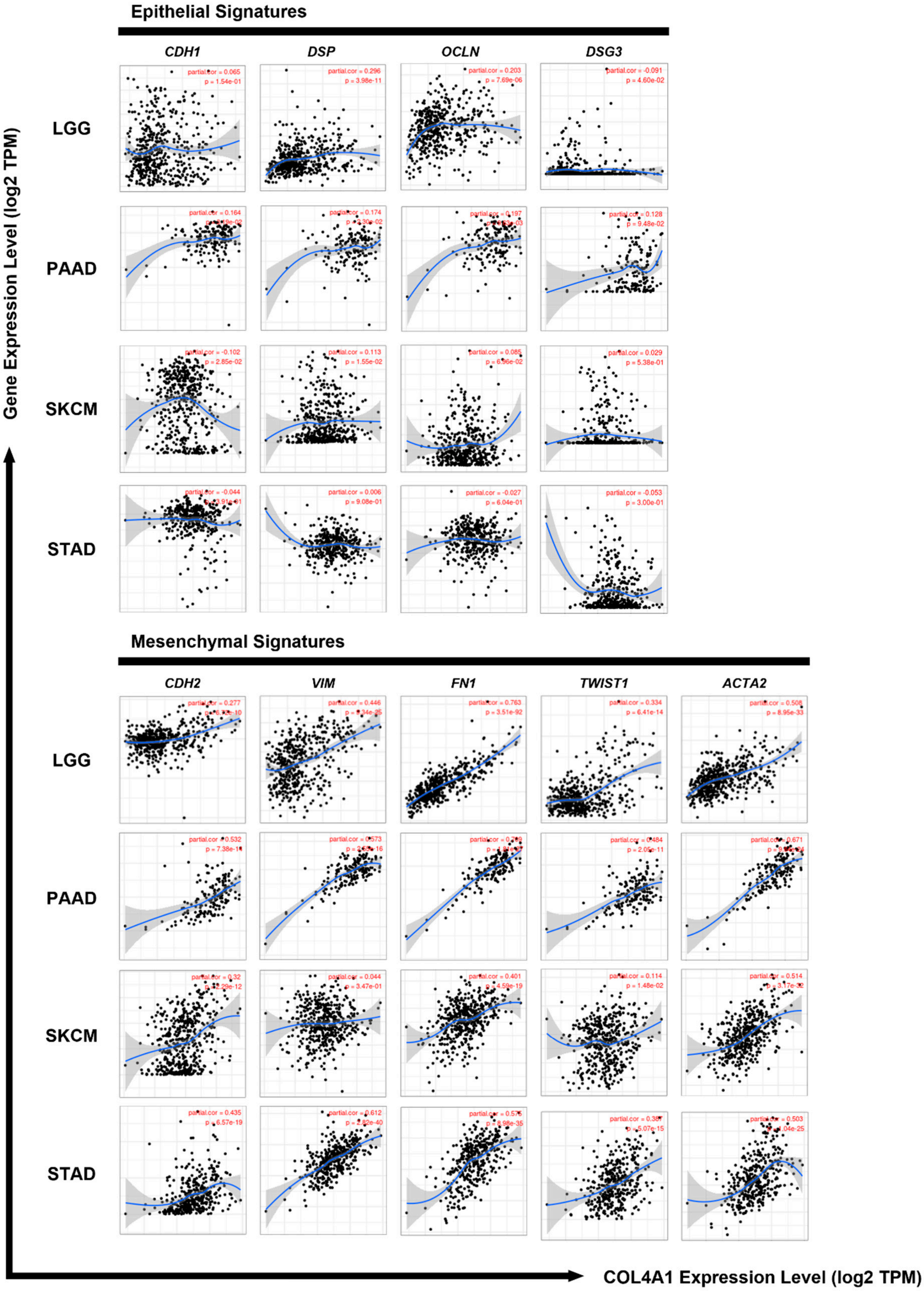

Figure S5. The correlation of *COL4A1* expression with the expression levels of various EMT signature genes in LGG, PAAD, SKCM, and STAD. Correlation analysis of individual type of cancers were retrieved from TIMER tool. Cadherin 1 (*CDH1*, E-cadherin), desmoplakin (*DSP*), occludin (*OCLN*),

and desmoglein 3 (*DSG3*) were used for epithelial signature genes; Cadherin 2 (*CDH2*, N-cadherin), vimentin (*VIM*), fibronectin 1 (*FN1*), twist family bHLH transcription factor (*TWIST1*), and alpha smooth muscle actin (*ACTA2*) were used for mesenchymal signatures.

Table S1. List of The Cancer Genome Atlas (TCGA) abbreviations for different types of cancer.

Abbreviation	Name
ACC	Adrenocortical carcinoma
BLCA	Bladder urothelial carcinoma
BRCA	Breast invasive carcinoma
CESC	Cervical squamous cell carcinoma and endocervical adenocarcinoma
CHOL	Cholangiocarcinoma
COAD	Colon adenocarcinoma
DLBC	Lymphoid neoplasm diffuse large B-cell lymphoma
ESCA	Esophageal carcinoma
GBM	Glioblastoma multiforme
HNSC	Head and neck squamous cell carcinoma
KICH	Kidney chromophobe
KIRC	Kidney renal clear cell carcinoma
KIRP	Kidney renal papillary cell carcinoma
LAML	Acute myeloid leukemia
LGG	Low-grade glioma
LIHC	Liver hepatocellular carcinoma
LUAD	Lung adenocarcinoma
LUSC	Lung squamous cell carcinoma
MESO	Mesothelioma
OV	Ovarian serous cystadenocarcinoma
PAAD	Pancreatic adenocarcinoma
PCPG	Pheochromocytoma and Paraganglioma
PRAD	Prostate adenocarcinoma
READ	Rectum adenocarcinoma
SARC	Sarcoma
SKCM	Skin cutaneous melanoma
STAD	Stomach adenocarcinoma
TGCT	Testicular germ cell tumors
THCA	Thyroid carcinoma
THYM	Thymoma
UCEC	Uterine corpus endometrial carcinoma
UCS	Uterine carcinosarcoma
UVM	Uveal melanoma

Table S2. Correlation analyses among the expression levels of collagen type IV alpha chain 1 (*COL4A1*) and related marker genes of T, B, monocytes, natural killer (NK), dendritic, Th2, Tfh, Th17, and exhausted T cells in low-grade glioma (LGG), pancreatic adenocarcinoma (PAAD), skin cutaneous melanoma (SKCM), and stomach adenocarcinoma (STAD)-TCGA datasets using the Tumor Immune Estimation Resource (TIMER) web tool.

Description	Gene Markers	LGG				PAAD				SKCM				STAD			
		None		Purity		None		Purity		None		Purity		None		Purity	
		Cor	*P*	Cor	*P*	Cor	*P*	Cor	*P*	Cor	*P*	Cor	P	Cor	*P*	Cor	*P*
T cells (general)	***CD3D***	0.382	***	0.427	***	0.322	***	0.253	***	-0.006	0.901	-0.118	*	0.093	0.059	0.069	0.182
	CD3E	0.403	***	0.438	***	0.358	***	0.29	***	0.009	0.842	-0.095	*	0.127	**	0.109	*
	CD2	0.448	***	0.477	***	0.367	***	0.292	***	0.021	0.654	-0.078	0.097	0.141	**	0.125	*
B cells	***CD19***	0.365	***	0.36	***	0.232	**	0.179	*	0.005	0.907	-0.059	0.207	0.147	**	0.134	**
	CD79A	0.2	***	0.208	***	0.275	***	0.213	**	-0.05	0.28	-0.133	**	0.108	*	0.077	0.135
Monocytes	***CD86***	0.262	***	0.303	***	0.611	***	0.562	***	0.218	***	0.188	***	0.284	***	0.271	***
	CSF1R	0.111	*	0.141	**	0.581	***	0.524	***	0.317	***	0.308	***	0.418	***	0.399	***
NK cells	***KIR2DL1***	0.203	***	0.204	***	0.22	**	0.219	**	0.003	0.949	-0.049	0.299	0.119	*	0.118	*
	KIR2DL3	0.274	***	0.288	***	0.279	***	0.291	***	0.037	0.42	-0.029	0.542	0.081	0.099	0.07	0.174
	KIR2DL4	0.249	***	0.243	***	0.15	*	0.164	*	-0.045	0.332	-0.127	**	-0.061	0.214	-0.087	0.089
	KIR3DL1	0.168	***	0.171	***	0.204	**	0.187	*	0.042	0.367	-0.015	0.751	0.085	0.085	0.06	0.242
	KIR3DL2	0.178	***	0.19	***	0.223	**	0.174	*	0.045	0.329	-0.029	0.533	0.122	*	0.128	*
	KIR3DL3	0.013	0.771	0.017	0.707	0.194	**	0.144	0.06	-0.061	0.183	-0.087	0.062	-0.029	0.555	-0.008	0.878
	KIR2DS4	0.188	***	0.213	***	0.047	0.531	0.053	0.487	0.055	0.23	0.005	0.914	0.102	*	0.099	0.055
	KLRK1	-0.083	0.059	-0.106	*	0.22	**	0.182	*	-0.001	0.988	-0.074	0.116	0.112	*	0.089	0.085
	NCR1	0.24	***	0.228	***	0.364	***	0.405	***	0.147	**	0.105	*	0.161	**	0.16	**
	NCR2	0.05	0.254	0.045	0.325	-0.098	0.193	-0.091	0.237	0.048	0.296	0.025	0.593	-0.089	0.07	-0.1	0.053
	NCR3	0.07	0.113	0.084	0.065	0.173	*	0.129	0.094	0.092	*	0.023	0.621	0.106	*	0.098	0.056
Dendritic cells	***HLA-DPB1***	0.444	***	0.472	***	0.471	***	0.412	***	0.112	*	0.052	0.27	0.135	**	0.117	*
	HLA-DQB1	0.392	***	0.415	***	0.392	***	0.335	***	0.101	*	0.046	0.329	0.1	*	0.079	0.124

	HLA-DRA	0.465	***	0.491	***	0.549	***	0.498	***	0.114	*	0.056	0.229	0.1	*	0.088	0.088
	HLA-DPA1	0.478	***	0.508	***	0.548	***	0.503	***	0.079	0.087	0.014	0.768	0.094	0.056	0.078	0.131
	CD1C	0.278	***	0.271	***	0.283	***	0.227	**	0.127	**	0.083	0.076	0.186	***	0.169	***
	NRP1	0.616	***	0.604	***	0.741	***	0.716	***	0.67	***	0.665	***	0.662	***	0.638	***
	ITGAX	0.219	***	0.262	***	0.497	***	0.434	***	0.175	***	0.128	**	0.395	***	0.381	***
**Th2**	***GATA3***	0.379	***	0.411	***	0.264	***	0.238	**	0.13	**	0.065	0.168	0.156	**	0.143	**
	STAT6	0.185	***	0.295	***	0.183	*	0.165	*	0.095	*	0.101	*	0.206	***	0.203	***
	STAT5A	0.28	***	0.32	***	0.361	***	0.313	***	-0.104	*	-0.109	*	0.312	***	0.299	***
	IL13	-0.022	0.62	-0.008	0.855	0.04	0.595	0.049	0.525	0.02	0.658	0.002	0.965	0.123	*	0.148	**
**Tfh**	***BCL6***	0.084	0.058	0.052	0.252	0.417	***	0.389	***	0.136	**	0.119	*	0.389	***	0.351	***
	IL21	0.098	*	0.082	0.073	0.156	*	0.134	0.081	0.044	0.336	0.003	0.943	0.009	0.858	0.003	0.956
**Th17**	***STAT3***	0.569	***	0.555	***	0.619	***	0.589	***	0.26	***	0.246	***	0.459	***	0.435	***
	IL17A	0.07	0.114	0.055	0.226	0.097	0.197	0.074	0.335	-0.035	0.445	-0.045	0.342	0.032	0.518	0.035	0.494
**Exhausted T cells**	***PDCD1***	0.409	***	0.42	***	0.283	***	0.209	**	-0.032	0.484	-0.133	**	0.13	**	0.127	*
	CTLA4	0.264	***	0.264	***	0.356	***	0.29	***	0.081	0.078	0.026	0.581	0.182	***	0.177	***
	LAG3	0.248	***	0.247	***	0.219	**	0.185	*	-0.03	0.518	-0.122	**	0.1	*	0.078	0.129
	HAVCR2	0.295	***	0.347	***	0.618	***	0.568	***	0.211	***	0.179	***	0.3	***	0.284	***

Asterisks denote the significance level of the correlation between the expression levels of *COL4A1* and each marker gene (* $P < 0.05$, ** $P < 0.01$, *** $P < 0.001$).

Table S3. Correlation analyses among the expression levels of *COL4A1* and related marker genes of the tumor microenvironment (TME) immune cells of four tumor groups from different origins using the R2 tool.

Description	Gene Markers	Tumor Glioma (GSE43378, n = 50)		Mixed Tumor Pancreas (GSE28735, n = 90)		Tumor Melanoma (GSE65904, n = 214)		Tumor Gastric (GSE15459, n = 192)	
		**r-value**	***p*-value**	**r-value**	***p*-value**	**r-value**	***p*-value**	**r-value**	***p*-value**
**CD8+ T cell**	***CD8A***	0.075	6.04×10^{-1}	-0.103	3.36×10^{-1}	-0.077	2.65×10^{-1}	-0.133	6.60×10^{-2}
	CD8B	-0.082	5.69×10^{-1}	0.116	2.74×10^{-1}	-0.143	**3.70×10^{-2}**	-0.123	9.00×10^{-2}
**T cells (general)**	***CD3D***	0.290	**4.10×10^{-2}**	0.320	**2.08×10^{-3}**	-0.004	9.59×10^{-1}	-0.077	2.89×10^{-1}
	CD3E	-0.111	4.43×10^{-1}	0.251	**1.70×10^{-2}**	0.049	4.75×10^{-1}	0.010	8.86×10^{-1}
	CD2	0.329	**2.00×10^{-2}**	0.353	**6.41×10^{-4}**	0.003	9.67×10^{-1}	-0.028	6.97×10^{-1}
**B cells**	***CD19***	-0.218	1.28×10^{-1}	-0.284	**6.64×10^{-3}**	-0.014	8.39×10^{-1}	-0.168	**2.00×10^{-2}**
	CD79A	-0.145	3.14×10^{-1}	0.099	3.53×10^{-1}	0.010	8.79×10^{-1}	-0.145	**4.40×10^{-2}**
**Monocytes**	***CD86***	0.412	**2.97×10^{-3}**	0.674	**3.49×10^{-13}**	0.162	**1.80×10^{-2}**	0.205	**4.43×10^{-3}**
	CSF1R	0.111	4.42×10^{-1}	0.686	**8.11×10^{-14}**	0.478	**1.33×10^{-13}**	0.419	**1.41×10^{-9}**
**TAMs**	***CCL2***	0.233	1.03×10^{-1}	0.254	**1.60×10^{-2}**	0.355	**9.09×10^{-8}**	0.452	**4.78×10^{-11}**
	CD68	N/A	N/A	N/A	N/A	0.208	**2.20×10^{-3}**	N/A	N/A
	IL10	0.305	**3.10×10^{-2}**	0.219	**3.80×10^{-2}**	0.201	**3.09×10^{-3}**	0.107	1.38×10^{-1}
**M1 macrophages**	***NOS2***	0.090	5.33×10^{-1}	-0.327	**1.69×10^{-3}**	0.118	8.50×10^{-2}	-0.190	**8.16×10^{-3}**
	IRF5	0.034	8.12×10^{-1}	0.207	5.00×10^{-2}	-0.026	7.03×10^{-1}	-0.051	4.85×10^{-1}
	PTGS2	0.328	**2.00×10^{-2}**	0.584	**1.55×10^{-9}**	0.362	**5.17×10^{-8}**	0.461	**1.75×10^{-11}**
**M2 macrophages**	***CD163***	0.777	**3.16×10^{-11}**	0.684	**1.06×10^{-13}**	0.375	**1.50×10^{-8}**	0.369	**1.43×10^{-7}**
	VSIG4	0.413	**2.88×10^{-3}**	0.624	**5.10×10^{-11}**	0.326	**1.05×10^{-6}**	0.307	**1.45×10^{-5}**
	MS4A4A	0.558	**2.52×10^{-5}**	0.521	**1.41×10^{-7}**	0.279	**3.46×10^{-5}**	0.290	**4.45×10^{-5}**
**Neutrophils**	***CEACAM8***	0.334	**1.80×10^{-2}**	0.248	**1.80×10^{-2}**	-0.015	8.26×10^{-1}	0.004	9.53×10^{-1}
	ITGAM	0.134	3.55×10^{-1}	0.768	**1.09×10^{-18}**	0.344	**2.42×10^{-7}**	0.278	**9.33×10^{-5}**

	CCR7	0.478	**4.50×10^{-4}**	0.370	**3.32×10^{-4}**	0.108	1.14×10^{-1}	0.003	9.67×10^{-1}
**NK cells**	***KIR2DL1***	0.032	8.27×10^{-1}	N/A	N/A	-0.067	3.33×10^{-1}	0.001	9.85×10^{-1}
	KIR2DL3	-0.370	**8.23×10^{-3}**	N/A	N/A	-0.143	3.70×10^{-2}	-0.153	**3.40×10^{-2}**
	KIR2DL4	-0.135	3.52×10^{-1}	N/A	N/A	-0.174	**1.10×10^{-2}**	-0.011	8.76×10^{-1}
	KIR3DL1	-0.156	2.81×10^{-1}	N/A	N/A	-0.052	4.53×10^{-1}	-0.046	5.26×10^{-1}
	KIR3DL2	N/A	N/A	N/A	N/A	-0.063	3.61×10^{-1}	N/A	N/A
	KIR3DL3	0.034	8.15×10^{-1}	N/A	N/A	-0.078	2.55×10^{-1}	-0.029	6.94×10^{-1}
	KIR2DS4	0.132	3.59×10^{-1}	N/A	N/A	-0.126	6.50×10^{-2}	-0.184	**1.10×10^{-2}**
	KLRK1	N/A	N/A	-0.019	8.58×10^{-1}	-0.060	3.81×10^{-1}	N/A	N/A
	NCR1	0.002	9.90×10^{-1}	-0.203	5.60×10^{-2}	-0.074	2.84×10^{-1}	-0.105	1.49×10^{-1}
	NCR2	-0.390	**5.16×10^{-3}**	-0.616	**1.02×10^{-10}**	-0.090	1.91×10^{-1}	-0.058	4.27×10^{-1}
	NCR3	0.078	5.90×10^{-1}	-0.389	**1.50×10^{-4}**	0.018	7.91×10^{-1}	-0.076	2.95×10^{-1}
**Dendritic cells**	***HLA-DPB1***	0.537	**5.74×10^{-5}**	0.572	**3.96×10^{-9}**	0.084	2.21×10^{-1}	0.023	7.55×10^{-1}
	HLA-DQB1	0.513	**1.39×10^{-4}**	0.612	**1.43×10^{-10}**	0.048	4.86×10^{-1}	-0.041	5.72×10^{-1}
	HLA-DRA	0.548	**3.83×10^{-5}**	0.659	**1.73×10^{-12}**	0.197	**3.74×10^{-3}**	-0.121	9.40×10^{-2}
	HLA-DPA1	0.457	**8.59×10^{-4}**	0.615	**1.18×10^{-10}**	0.124	7.00×10^{-2}	-0.023	7.52×10^{-1}
	CD1C	0.047	7.45×10^{-1}	0.445	**1.11×10^{-5}**	0.204	**2.67×10^{-3}**	0.097	1.80×10^{-1}
	NRP1	0.722	**3.25×10^{-9}**	0.760	**3.49×10^{-18}**	0.704	**2.62×10^{-33}**	0.577	**1.82×10^{-18}**
	ITGAX	0.315	**2.60×10^{-2}**	0.698	**2.11×10^{-14}**	0.096	1.62×10^{-1}	0.331	**2.75×10^{-6}**
**Th1**	***TBX21***	0.323	**2.20×10^{-2}**	-0.487	**1.16×10^{-6}**	-0.240	**3.91×10^{-4}**	-0.066	3.64×10^{-1}
	STAT4	-0.542	**4.72×10^{-5}**	0.294	**4.89×10^{-3}**	0.081	2.38×10^{-1}	0.069	3.42×10^{-1}
	STAT1	0.478	**4.43×10^{-4}**	0.294	**4.89×10^{-3}**	-0.167	**1.40×10^{-2}**	-0.218	**2.38×10^{-3}**
	IFNG	0.338	**1.60×10^{-2}**	0.095	3.71×10^{-1}	-0.184	**6.87×10^{-3}**	-0.086	2.35×10^{-1}
	TNF	-0.352	**1.20×10^{-2}**	-0.205	**5.30×10^{-2}**	0.048	4.82×10^{-1}	0.043	5.57×10^{-1}
**Th2**	***GATA3***	0.385	**5.79×10^{-3}**	0.426	**2.83×10^{-5}**	-0.013	8.54×10^{-1}	0.034	6.36×10^{-1}
	STAT6	0.103	4.78×10^{-1}	0.698	**1.96×10^{-14}**	-0.012	8.59×10^{-1}	0.156	**3.10×10^{-2}**

	STAT5A	0.121	4.03×10^{-1}	0.619	**8.00×10^{-11}**	0.064	3.54×10^{-1}	0.184	**1.10×10^{-2}**
	IL13	0.072	6.20×10^{-1}	-0.207	5.00×10^{-2}	-0.065	3.41×10^{-1}	-0.123	9.00×10^{-2}
**Tfh**	***BCL6***	0.375	**7.27×10^{-3}**	0.629	**3.04×10^{-11}**	0.433	**3.48×10^{-11}**	0.412	**2.77×10^{-9}**
	IL21	0.065	6.52×10^{-1}	-0.316	**2.41×10^{-3}**	-0.075	2.73×10^{-1}	-0.099	1.74×10^{-1}
**Th17**	***STAT3***	0.548	**3.82×10^{-5}**	0.785	**5.09×10^{-20}**	0.509	**1.73×10^{-15}**	0.266	1.89×10^{-4}
	IL17A	-0.075	6.05×10^{-1}	-0.343	**9.45×10^{-4}**	-0.235	**5.21×10^{-4}**	-0.021	7.70×10^{-1}
**Treg**	***FOXP3***	-0.299	**3.50×10^{-2}**	-0.164	1.24×10^{-1}	0.005	9.39×10^{-1}	-0.147	**4.20×10^{-2}**
	CCR8	0.117	4.20×10^{-1}	0.343	**9.27×10^{-4}**	-0.047	4.90×10^{-1}	-0.113	1.20×10^{-1}
	STAT5B	-0.112	4.38×10^{-1}	0.620	**7.18×10^{-11}**	0.199	**3.39×10^{-3}**	0.259	**2.78×10^{-4}**
	TGFB1	0.107	4.59×10^{-1}	0.855	**6.98×10^{-27}**	-0.019	7.81×10^{-1}	0.445	**1.01×10^{-10}**
**Exhausted T cells**	***PDCD1***	0.211	1.41×10^{-1}	-0.040	7.11×10^{-1}	-0.107	1.18×10^{-1}	-0.064	3.77×10^{-1}
	CTLA4	-0.149	3.02×10^{-1}	0.509	**3.02×10^{-7}**	-0.176	**9.68×10^{-3}**	-0.012	8.69×10^{-1}
	LAG3	-0.152	2.92×10^{-1}	-0.403	**8.17×10^{-5}**	-0.178	**9.02×10^{-3}**	-0.025	7.31×10^{-1}
	HAVCR2	0.253	7.60×10^{-2}	0.664	**9.43×10^{-13}**	0.194	**4.46×10^{-3}**	0.232	**1.21×10^{-3}**

Bold values indicate the statistical significance at $p < 0.05$.
